# Clinical impact of fetal sac size on closed neural tube defects

**DOI:** 10.1007/s00381-025-06866-6

**Published:** 2025-07-09

**Authors:** Sierra D. Land, Taryn Gallagher, Sanjana R. Salwi, Tom A. Reynolds, Leny Mathew, Edward R. Oliver, Dana A. Weiss, Tracy M. Flanders, Juliana S. Gebb, N. Scott Adzick, Gregory G. Heuer

**Affiliations:** 1 Richard D. Wood Jr. Center for Fetal Diagnosis and Treatment, Children’s Hospital of Philadelphia, Philadelphia, PA USA; 2 Division of Neurosurgery, Department of Surgery, Children’s Hospital of Philadelphia, Philadelphia, PA USA; 3 Department of Radiology, Children’s Hospital of Philadelphia, Philadelphia, PA USA; 4 Division of Urology, Department of Surgery, Children’s Hospital of Philadelphia, Philadelphia, PA USA; 5 Division of Pediatric General, Thoracic and Fetal Surgery, Children’s Hospital of Philadelphia, Philadelphia, PA USA

**Keywords:** Neural tube defects, Lipomyelomeningocele, Myelocystocele, Tethered cord

## Abstract

**Purpose:**

To evaluate the association between lesion volume and postnatal outcomes in patients with closed spina bifida (SB).

**Methods:**

Single-center retrospective review of all patients diagnosed with a closed SB evaluated between 2013 and 2023. Prenatal lesion volume < 3 mL was categorized as “no sac,” and volume ≥ 3 mL was categorized as “sac.”

**Results:**

Of eligible patients, 22 had a sac, and 33 did not. Myelocystocele patients more commonly had an associated saccular lesion than lipomyelomeningoceles (80% vs. 28%, *p* = 0.02). Lipomyelomeningocele patients demonstrated less prenatal lesion growth compared to myelocystocele patients (6.26 vs. 58.0 mL) over a median of 12.7 and 10.5 weeks, respectively. Patients with a sac had a higher proportion of talipes (40.9% vs. 9.1%; *p* = 0.007) compared to those without. At 5 years old, a higher proportion of patients with no sac was able to ambulate independently and required less CIC than patients with a sac (80% vs. 42%; 0% vs. 41.7%). VUDS showed a greater frequency of detrusor overactivity (60.0% vs. 45.0%) and abnormal bladder compliance (60.0% vs. 5.0%) in those with a sac compared to those without. Repeat tethered cord release was also more common (30.0% vs. 3.4%) among patients with a sac-associated defect.

**Conclusions:**

Patients with a sac had an increased risk of prenatal talipes, decreased ability to ambulate independently, increased use of CIC, and increased need for repeat tethered cord release compared to patients without a sac. Given numerous associated morbidities reported in this study, longitudinal multidisciplinary follow-up is critical in the care of these patients.

## Introduction

Neural tube defects (NTDs) compose a spectrum of structural birth defects estimated to impact 1–5 per 1000 live births globally [1, 2]. The most common form of NTDs is spina bifida (SB), caused by a failure of vertebral arch formation, resulting in an opening in the vertebral column [3, 4]. Most SB cases are classified as “open,” in which the neural tissue is exposed to amniotic fluid with or without a meninges-based sac (myelomeningocele or myeloschisis) [5–7]. Ten to 15% of cases are classified as “closed” in which vertebral arches are incomplete but the neural tube is covered by skin or fat [7, 8]. Closed SB varies widely in clinical presentation, and long-term outcome reports among these patients are limited [9–11].

Substantial research has demonstrated associations between prenatal characteristics and postnatal outcomes in patients with open SB [12–16]. Recent publications have shown an association between the presence of sac and a higher incidence of talipes as well as an inverse relationship between sac size and motor function [17, 18].

This study aims to perform a detailed analysis of the sac size and volume in patients with closed neural tube defects. We studied the in utero growth of these lesions and the relationship between sac volume and functional outcomes in patients with closed SB.

## Methods

This was a retrospective review of all patients with a prenatal diagnosis of SB evaluated at a single level III fetal center between January 2013 and December 2023. Patients included in the study had a confirmed postnatal diagnosis of closed SB, defined as a fat or fully skin-covered defect. Patients with open SB, such as myelomeningocele or myeloschisis, were excluded from the study.

All data was abstracted from the electronic medical record (EMR). A detailed anatomical ultrasound was interpreted by a fetal radiologist to assess the level of the osseous defect, cerebral ventricular atrial diameters (ADs), and SB defect measurements. Lesion measurements were obtained in three orthogonal planes with the defect positioned away from the uterine wall and placenta. Lesion volumes were calculated from reported measurements using the formula of a prolate ellipsoid (length × height × width × 0.52). Prenatal AD was reported as the largest transverse diameter of the two ventricles in millimeters (mm). All prenatal ultrasound scans, interpreted by either a fetal radiologist or a maternal fetal medicine specialist, were reviewed for the presence or absence of talipes. Neurological magnetic resonance imaging (MRI) was used to assess for hindbrain herniation, with interpretation by a pediatric neuroradiologist.

Ambulation, urinary, and bowel characteristics were assessed by a physical therapist, specialized pediatrician, and urologist within the interdisciplinary SB Program. The follow-up at age 2 years and 5 years was defined as the appointment closest to 24 and 60 months of age, respectively.

Video urodynamic studies (VUDSs) were performed and reviewed by a pediatric urologist. VUDS used a catheter to fill the bladder at a fixed rate while measuring pressure in combination with fluoroscopic imaging. Patients with significant bladder surgery history were not included in the VUDS analysis. The VUDS closest to 24 months old was assessed for pressure, compliance, capacity, and detrusor overactivity. Pressure at actual capacity was defined as the pressure measured immediately prior to voiding, leak, or end of study. Compliance was defined as the change of bladder volume over the change in detrusor pressure during filling (*C* = Δ*V*/ΔPdet). Abnormal compliance is a subjective measure defined as an observed continual increase in bladder pressure during filling, whereas bladders with normal compliance maintain low pressure throughout filling. The percent bladder capacity was calculated by dividing total milliliters (mL) within the bladder just prior to voiding, leak, or end of study by the patient’s expected bladder capacity (weight (kg) × 7). Studies were also evaluated for the presence or absence of detrusor overactivity, defined as periodic short-duration contractions of the detrusor muscle.

To ensure complete surgical history, only patients with initial defect repair at the institution of study were included. Wound revision was defined as a reoperation for wound dehiscence following initial defect repair. Repeat tethered cord release was indicated for patients with a decline in lower-extremity function, pain, or worsening neurogenic change in bladder/bowel function. CSF diversion was defined as placement of ventriculoperitoneal (VP) shunt.

Sac presence was determined based on a visual inspection of the distribution of lesion volume. Since there was a clear delineation of patients by volume at 3 mL, we categorized patients with a prenatal lesion volume < 3 mL as no sac and volume ≥ 3 mL as having sac.

Continuous variables were summarized as median (25 th percentile [Q1], 75 th percentile [Q3]). Categorical data were summarized as frequencies. The Fisher exact and Mann–Whitney *U* tests were used to evaluate the association between the presence of sac with prenatal and demographic characteristics. Unadjusted relative risk and 95% confidence interval (CI) were calculated from the tables. Given the small sample size and descriptive nature of this study, no formal statistical comparisons were performed on the functional outcome metrics. All tables, figures, and statistical analyses were completed using RStudio 2024.04.01 on R version 4.3.2 [19]. The study was approved by the local Institutional Review Board, and all data were collected, validated, and stored using the Clinical Outcomes Data Archive [20].

## Results

**Fig. 1:**
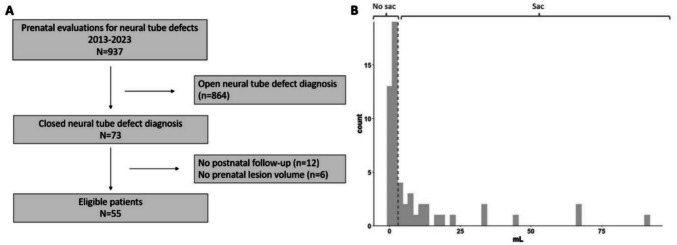
**A** Histogram of lesion volume and **B** cohort diagram

**Fig. 2:**
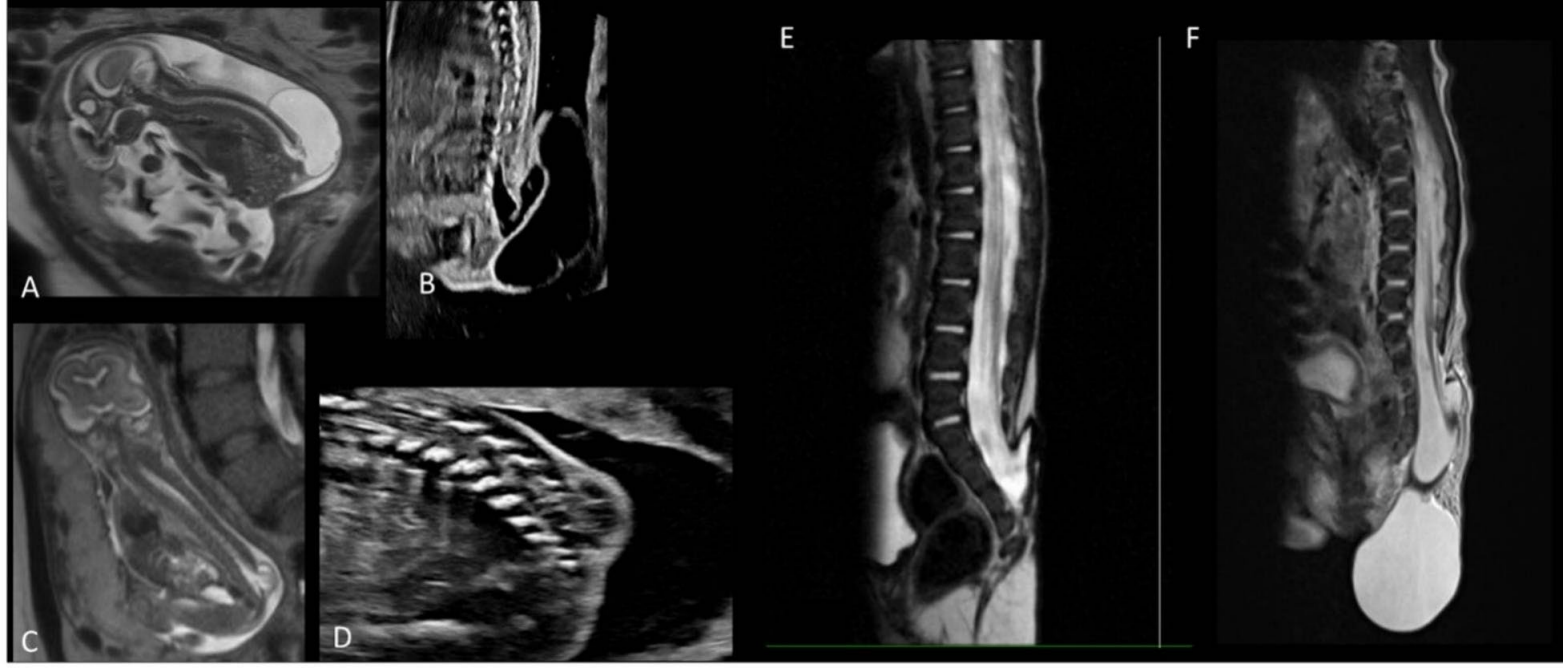
Imaging of sac and no sac defects. **A** Prenatal MRI of 21-week fetus with myelocystocele (sac) defect. **B** Prenatal ultrasound of the sagittal spine of the same fetus from image **A**. **C** Prenatal MRI of 21-week fetus with lipomyelomeningocele (no sac) defect. **D** Prenatal ultrasound of the sagittal spine of the same fetus from image **C**. **E** Postnatal MRI of 3-month-old with meningocele (no sac) defect. **F** Postnatal MRI of 1-day-old with meningocele (sac) defect

Between January 2013 and December 2023, 937 patients underwent a prenatal evaluation for SB, of which 864 and 73 were found to have open and closed SB, respectively. Of the closed cases, 55 patients had at least one assessment of prenatal lesion volume with postnatal follow-up (Fig. 1A). Thirty-three patients had an initial lesion volume < 3 mL (no sac) and 22 patients ≥ 3 mL (sac) (Fig. 1B). Prenatal imaging examples of sac vs. no sac patients are shown in Fig. 2A–D. As reported in Table 1, both groups were a majority non-Hispanic/White. Patients with no sac presented at an earlier median gestational age (22.5 weeks) compared to those with a sac (24.2 weeks; *p* = 0.025). Patients without a sac had lesion volumes ranging from a minimum of 0.07 to a maximum of 2.97 mL, whereas patients with a sac had volumes between 4.24 and 91.0 mL (*p* < 0.001). Over half the patients in this study had a lipomyelomeningocele; however, the frequency of each diagnosis varied based on sac presence. Myelocystocele patients more commonly had an associated saccular lesion than lipomyelomeningoceles (36.4% vs. 18.2%). Osseous defect level, AD, and hindbrain herniation did not vary between groups, with most patients having low-level defects without ventriculomegaly or hindbrain herniation. However, compared to no sac, patients with a sac had a significantly higher proportion of talipes (40.9% vs. 9.1%; *p* = 0.007; unadjusted relative risk 2.5, 95% CI 1.42–4.34).

**Table 1:** Demographic and prenatal characteristics by sac presence

	No Sac (*N*=33)	Sac (*N*=22)	Overall *N*=55)	*p***-**value
Gestational age at evaluation				
Median [Q1, Q3]	22.5 [21.5, 24.3]	24.2 [22.9, 27.3]	23.0 [21.9, 25.4]	0.025*
Lesion volume (mL)				
Median [Q1, Q3]	1.32 [0.749, 2.20]	11.7 [6.92, 30.9]	2.25 [1.05, 8.78]	< 0.001*
Race				
Non-White	10 (30.3%)	3 (13.6%)	13 (23.6%)	0.203
White	23 (69.7%)	19 (86.4%)	42 (76.4%)	
Ethnicity				
Not Hispanic or Latino	26 (78.8%)	20 (90.9%)	46 (83.6%)	0.289
Hispanic or Latino	7 (21.2%)	2 (9.1%)	9 (16.4%)	
Diagnosis				
Lipomeningocele	23 (69.7%)	9 (40.9%)	32 (58.2%)	0.020*
Meningocele	7 (21.2%)	4 (18.2%)	11 (20.0%)	
Myelocystocele	2 (6.1%)	8 (36.4%)	10 (18.2%)	
Other	1 (3.0%)	1 (4.5%)	2 (3.6%)	
Lesion level				
T12 and above	5 (15.2%)	5 (22.7%)	10 (18.2%)	0.760
L1–L3	9 (27.3%)	6 (27.3%)	15 (27.3%)	
L4 and below	19 (57.6%)	11 (50.0%)	30 (54.5%)	
Prenatal atrial diameter (US)				
< 10 mm	30 (90.9%)	21 (95.5%)	51 (92.7%)	0.762
10–15 mm	2 (6.1%)	0 (0%)	2 (3.6%)	
> 15 mm	1 (3.0%)	1 (4.5%)	2 (3.6%)	
Hindbrain herniation				
No	33 (100%)	20 (90.9%)	53 (96.4%)	0.156
Yes	0 (0%)	2 (9.1%)	2 (3.6%)	
Prenatal talipes				
No	30 (90.9%)	13 (59.1%)	43 (78.2%)	0.007*
Yes	3 (9.1%)	9 (40.9%)	12 (21.8%)	

*Significant *p*-value ≤ 0.05

**Fig. 3:**
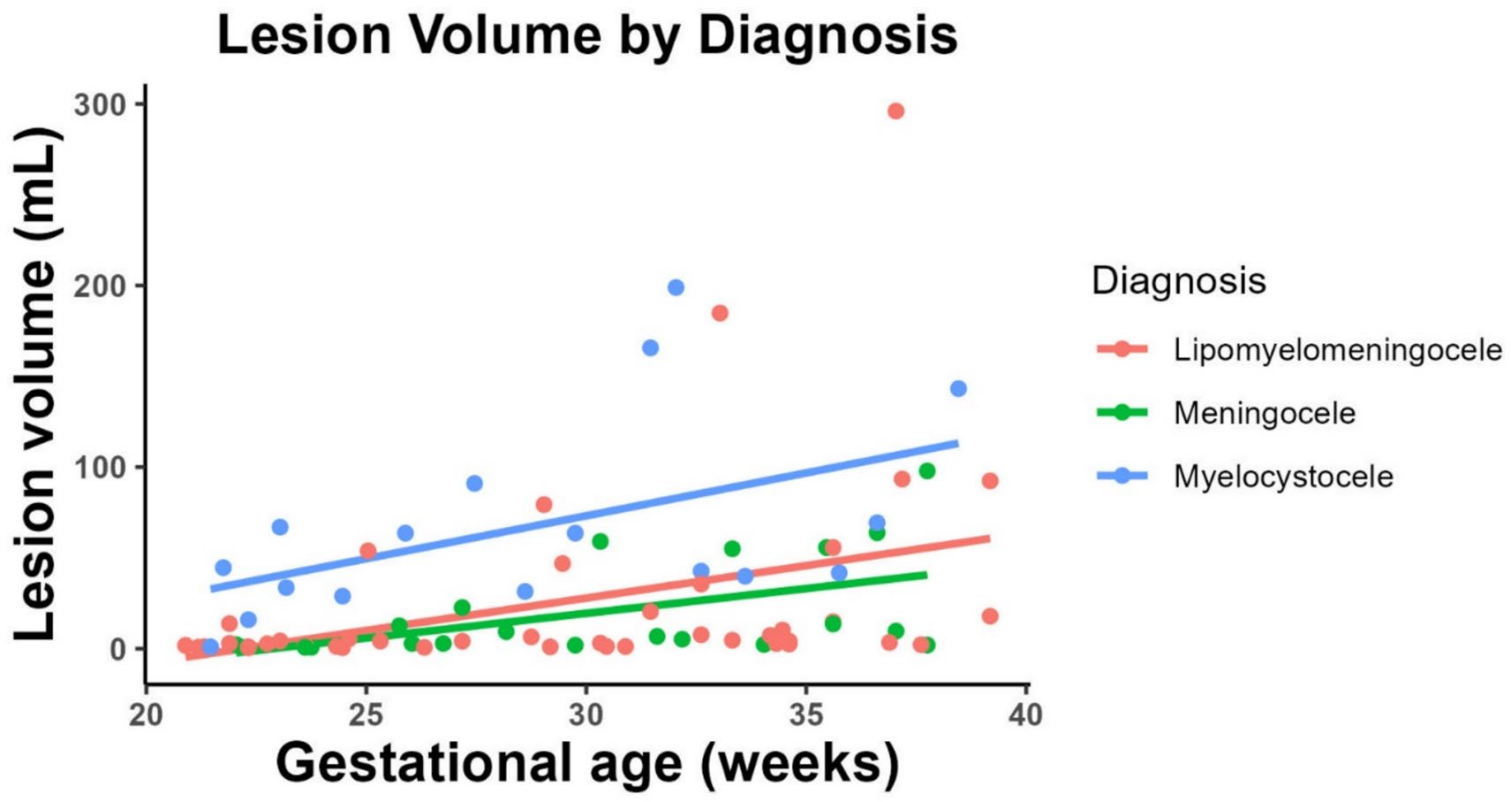
Prenatal lesion volume by diagnosis

A subanalysis was performed of patients with serial prenatal lesion volume measurements and is illustrated in Fig. 3. Among the 55 eligible patients, 29 (17 no sac, 12 sac) had at least two prenatal lesion volume assessments. Fifteen patients with a lipomyelomeningocele demonstrated a median growth of 6.26 [2.24, 13.8] mL over a median duration of 12.7 [10.1, 14.6] weeks. Whereas 6 patients with a myelocystocele had a median growth of 58.0 [13.3, 71.6] mL over 10.5 [5.07, 13.9] weeks. Eight meningocele patients were also included with a median growth of 1.77 [0.55, 19.9] mL over 9.15 [7.0, 11.9] weeks.

**Fig. 4:**
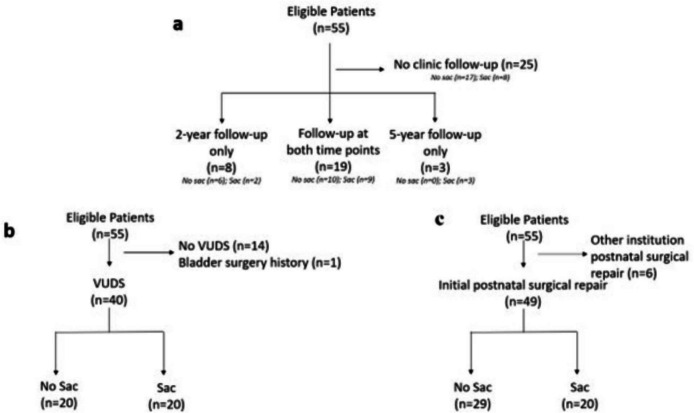
Follow-up cohort **A** spina bifida follow-up clinic. **B** VUDS cohort. **C** Surgical cohort

Table 2 describes ambulation, urinary, and bowel characteristics at 2 and 5 years of age stratified by sac presence. As outlined in Fig. 4A, 30 patients returned for follow-up evaluation during at least one timepoint with 19 patients evaluated at both timepoints (10 no sac, 9 sac). A higher proportion of patients with no sac was able to ambulate independently compared to those with a sac (56% vs. 27%; 80% vs. 42%) at both 2 and 5 years of age, respectively. At 2 years old, for patients who needed assistive devices, the braces or devices used were similar regardless of sac presence. However, at 5 years old, patients with no sac who required assistive devices only used supramalleolar (SMO) or ankle–foot orthosis (AFO). Patients with a sac utilized a wider range of braces and devices, including knee (KAFO) and hip (HKAFO) orthoses, crutches, walkers, and wheelchairs.

**Table 2:** Postnatal outcomes by sac presence

	2 years		5 years	
	No sac (*N* = 16)	Sac (*N* = 11)	No sac (*N* = 10)	Sac (*N* = 12)
Age (years)	2.08 [1.92, 2.26]	2.04 [1.97, 2.13]	4.60 [4.28, 4.66]	5.22 [4.78, 5.38]
Ambulation				
Independently	9 (56.3%)	3 (27.3%)	8 (80.0%)	5 (41.7%)
With assistive devices	5 (31.3%)	4 (36.4%)	2 (20.0%)	6 (50.0%)
Non-ambulator	1 (6.3%)	2 (18.2%)	0 (0%)	1 (8.3%)
Unable to assess due to age	1 (6.3%)	2 (18.2%)	0 (0%)	0 (0%)
Ambulation aids				
None	9 (56.3%)	3 (27.3%)	8 (80.0%)	5 (41.7%)
Yes^a^	7 (43.8%)	8 (72.7%)	2 (20.0%)	7 (58.3%)
*SMOs*	2 (12.5%)	2 (18.2%)	1 (10.0%)	0 (0%)
AFOs	5 (31.3%)	8 (72.7%)	1 (10.0%)	6 (50.0%)
KAFOs	0 (0%)	1 (9.1%)	0 (0%)	1 (8.3%)
HKAFOs	0 (0%)	0 (0%)	0 (0%)	1 (8.3%)
Crutches	0 (0%)	0 (0%)	0 (0%)	1 (8.3%)
Walker	3 (18.8%)	4 (36.4%)	0 (0%)	3 (25.0%)
Wheelchair	0 (0%)	1 (9.1%)	0 (0%)	3 (25.0%)
Urinary management				
None	12 (75.0%)	4 (36.4%)	9 (90.0%)	5 (41.7%)
Yes^a^	4 (25.0%)	7 (63.6%)	1 (10.0%)	7 (58.3%)
Clean intermittent catheterization	3 (18.8%)	6 (54.5%)	0 (0%)	5 (41.7%)
Bladder relaxant medications^*b*^	2 (12.5%)	4 (36.4%)	1 (10.0%)	4 (33.3%)
Bowel management				
None	9 (56.3%)	6 (54.5%)	6 (60.0%)	3 (25.0%)
Yes^a^	7 (43.8%)	5 (45.5%)	4 (40.0%)	9 (75.0%)
Laxatives	3 (18.8%)	5 (45.5%)	2 (20.0%)	7 (58.3%)
Dietary modification	3 (18.8%)	1 (9.1%)	3 (30.0%)	1 (8.3%)
Enemas	1 (6.3%)	0 (0%)	1 (10.0%)	3 (25.0%)
Colostomy	1 (6.3%)	0 (0%)	0 (0%)	0 (0%)

^a^Not mutually exclusive

^b^Bladder relaxant medications include oxybutynin, solifenacin, and tolterodine

Overall, urinary management was used more frequently in patients with a sac compared to those without. By 5 years of age, only one patient without a sac required any urinary management in the form of a bladder relaxant medication, whereas 7 (58.3%) of 5-year-old patients with a sac required urinary management interventions and 5 (41.7%) in the form of clean intermittent catheterizations. At 2 years of age, there was a similar proportion of patients using some form of bowel management among the two groups (43.8% no sac vs. 45.5% sac). By 5 years old, a higher proportion (*n* = 9, 75.0%) of patients with a sac required bowel management compared to those without (*n* = 4, 40.0%). Types of bowel management varied between the two cohorts as well, with patients in the sac cohort requiring pharmacological intervention with laxatives and enemas, while the most common bowel management technique in the no sac cohort was dietary modification.

Video urodynamic studies were used to assess functional bladder characteristics by sac presence (Table 3), with 40 patients having a complete study (Fig. 4B). Between the groups, there was no difference in the median age of patients at the time of the study. Patients with a sac had a slightly higher median pressure at bladder capacity (6.50 vs. 3.50 cmH2O) compared to those without. Most notably, 12 (60%) of patients with a sac were found to have abnormal bladder compliance compared to 1 (5%) of patients without a sac. In both groups, the median percent bladder capacity achieved was > 90%, with a slightly higher median percent capacity achieved in patients with a sac. Detrusor overactivity was common among both groups but was found more frequently in those with a sac compared to those without (*n* = 12, 60% vs. *n* = 9, 45%).

**Table 3:** Video urodynamic studies by sac presence

	No sac (*N* = 20)	Sac (*N* = 20)	Overall (*N* = 40)
Age at study (years)			
Median [Q1, Q3]	1.69 [0.895, 1.97]	1.66 [1.01, 2.14]	1.66 [0.952, 2.01]
Pressure at actual bladder capacity (cmH2O)			
Median [Q1, Q3]	3.50 [2.00, 5.00]	6.50 [4.75, 14.3]	5.00 [3.00, 9.25]
Percentage of estimated bladder capacity achieved			
Median [Q1, Q3]	92.9 [51.8, 112]	99.2 [74.6, 130]	98.8 [68.7, 114]
Bladder compliance			
Abnormal	1 (5.0%)	12 (60.0%)	13 (32.5%)
Normal	19 (95.0%)	8 (40.0%)	27 (67.5%)
Detrusor overactivity			
No	11 (55.0%)	8 (40.0%)	19 (47.5%)
Yes	9 (45.0%)	12 (60.0%)	21 (52.5%)

Forty-nine patients underwent the initial defect repair at the same institution as the prenatal evaluation (Fig. 4C). Postnatal MRI examples are shown in Fig. 2E, F. At the time of initial defect repair, the median age in patients without a sac was 3.3 months compared to less than 1 month in patients with a sac. Overall, the need for a repeat tethered cord release was low [7 (14.3%)] but was required more frequently in patients with a sac compared to those without [6 (30.0%) vs. 1 (3.4%)]. Nine patients required a wound revision following initial defect repair, of which 7 were patients without a sac. Only 5 patients had a CSF leak following defect repair (4 no sac; 1 sac). Overall, only 5 (10.2%) patients required permanent CSF diversion. A higher proportion of patients with a sac needed permanent CSF diversion compared to those without a sac [3 (15.0%) vs. 2 (6.9%)]. Among the 3 patients with a sac, CSF diversion was indicated in two cases for an enlarging head circumference and full fontanelle and in one case following a CSF leak after pseudomeningocele development. CSF diversion was indicated in both patients without a sac following a CSF leak, with one also developing a pseudomeningocele (Table 4).

**Table 4:** Surgical characteristics by sac presence

	No sac (*N* = 29)	Sac (*N* = 20)	Overall (*N* = 49)
Age at resection (months)			
Median [Q1, Q3]	3.33 [2.80, 4.23]	0.183 [0.0917, 3.16]	3.13 [0.167, 3.77]
Repeat tethered cord release			
No	28 (96.6%)	14 (70.0%)	42 (85.7%)
Yes	1 (3.4%)	6 (30.0%)	7 (14.3%)
Wound revision			
No	22 (75.9%)	18 (90.0%)	40 (81.6%)
Yes	7 (24.1%)	2 (10.0%)	9 (18.4%)
CSF leak			
No	25 (86.2%)	19 (95.0%)	44 (89.8%)
Yes	4 (13.8%)	1 (5.0%)	5 (10.2%)
CSF diversion			
No	27 (93.1%)	17 (85.0%)	44 (89.8%)
Yes	2 (6.9%)	3 (15.0%)	5 (10.2%)
Reason for CSF diversion			
CSF leak	1 (3.4%)	0 (0%)	1 (2.0%)
CSF leak + pseudomeningocele	1 (3.4%)	1 (5.0%)	2 (4.1%)
Enlarging head circumference + full fontanelle	0 (0%)	2 (10.0%)	2 (4.1%)
No CSF diversion	27 (93.1%)	17 (85.0%)	44 (89.8%)

## Discussion

Previous studies have demonstrated the association between prenatal lesion volume and functional outcomes in patients with open SB [17, 18]. However, to our knowledge, this is the first study examining the relationship in patients with closed SB. This single-center study demonstrates that despite similar osseous defect levels, patients with a sac had a significantly increased risk of prenatal talipes, decreased ability to ambulate independently, increased use of CIC, and increased need for repeat tethered cord release compared to patients without a sac. In addition, this study reports similar findings of detrusor overactivity, bladder capacity, and need for permanent CSF diversion regardless of sac presence.

Consistent with prior studies, few patients were observed to have prenatal hindbrain herniation or ventriculomegaly [10, 11]. Compared to open SB, the skin/fat covering over the neural tube in closed SB prevents CSF leakage, allowing for normal positioning of cerebellar tissue and unobstructed CSF flow [2, 21]. The observed difference in diagnosis between the groups is largely due to the etiology of each diagnosis. In patients with myelocystoceles, the inadequate regression of the central canal leads to an enlargement of the terminal cystic spinal cord. As the cyst expands, it pushes beyond the thecal sac and creates a “double cyst” of the ballooned spinal cord and meningocele [2, 11]. In lipomyelomeningoceles, premature disjunction creates contact between the mesenchyme and the developing neural tube, inducing fat formation. However, the extent of fatty tissue formation is limited laterally as the ventral surface of the neural plate induces the mesenchyme to form meninges [10, 22]. While pure cases of myelocystocele or lipomyelomeningocele occur, many cases lie somewhere along a spectrum between the two pathologies. Therefore, we speculate that it may be more useful to consider the size of the defect rather than just diagnosis alone when predicting outcomes in these patients.

Congenital talipes equinovarus, or clubfoot, is a known associated anomaly in children with NTDs. It is observed in approximately 20–40% of patients with open SB, whereas the frequency in patients with closed SB is largely unreported [9, 23–25]. Closed SB patients, with or without a sac, are vulnerable to potential neurological damage due to the initial failure of neural tube closure; however, the closed nature of the defects protects patients against the chemotoxicity of the uterine environment [26]. Oliver et al. proposed that open SB cases with a sac may be subjected to additional mechanical neurologic injury caused by stretching of the neural placode and spinal nerves within the sac, leading to worsening functional outcomes. Therefore, despite similar osseous defect levels, the higher rate of prenatal talipes in patients with a sac supports the previously purposed theory that traction on the spinal cord and spinal nerves during the development and growth of the sac causes additional neurologic injury [17, 18].

Regardless of sac presence, the associated defect prevents the upward mobility of the conus medullaris during normal fetal and childhood development. After birth, elective surgery is recommended to free the spinal cord and nerves from the associated defect, avoiding progressive neurological dysfunction. Consistent with prior literature, we observed low rates of complications (wound revision and CSF leak) following repair during early infancy [9–11]. Patients without a sac required a wound revision at a slightly higher proportion than those with a sac. This is most likely due to no sac patients having less skin associated with the defects, resulting in higher closure tension, therefore increasing the frequency of wound dehiscence. While most patients can experience improvement in functional grade, not all neurologic damage caused during embryologic formation can be reversed by the operation [10, 27]. Long-term outcomes following surgery in patients with closed SB are variable. One study reports 11.8% of myelocystocele patients were non-ambulating and 59% used assistive devices at the time of follow-up [11], while another found 48% of lipomyelomeningcele patients had an abnormal lower-extremity neurological examination during follow-up [28]. In our study, we observed 36% of patients needing braces/assistive devices, and only 4% were non-ambulatory at 5 years of age. Differences in outcomes between studies may be attributed to variability in cohort composition and patients’ ages at follow-up.

Patients with open SB have decreased peripheral nerve innervation and a lower number of nerve fibers within the bladder, believed to be a significant cause of neurogenic bladder in this population [29, 30]. The majority of studies have observed no significant change in bladder function in open SB patients receiving fetal repair compared to postnatal closure [15, 31, 32]. Therefore, injury to the nerves of the bladder may largely result from the initial abnormal spinal cord formation and mechanical injury from the growth of the sac rather than amniotic fluid exposure. This mechanism may also explain neurogenic bladder findings in patients with closed SB. In our study, more patients with a sac required intervention to manage bowel/bladder function and CIC than patients without a sac at both time points. VUDS demonstrated decreased normal bladder compliance in patients with a sac, while patients without a sac had mostly normal VUDS. These findings support the theory of increased neurological injury associated with the growth of the sac and highlight the importance of urology consultation in the management of these patients.

### Limitations

Generalizability of this study is limited by its single-center retrospective nature and small sample size. Outcomes may reflect management specific to our institution and not be representative of broader clinical practices. Additional variance could be introduced due to changes in clinical practice and medical record documentation over the study period. All SB patients are offered recommended follow-up appointments within the Spina Bifida Clinic; however, patients may elect to receive follow-up care at another institution. Therefore, long-term outcomes are potentially confounded by unaccounted-for characteristics of patients that elect to return for recommended follow-up. Eighteen patients (11 no sac, 7 sac) were not eligible for 5-year follow-up assessment as they had not yet reached the appropriate age. Of those, seven were also not eligible for 2-year assessment as they were too young. Given the limited sample size, this study was not powered to conduct formal confounder-adjusted statistical analysis on outcome metrics. The greatest strength of this study was the ability to describe and compare granular data, from prenatal characteristics to outcomes at 5 years of age, among a cohort of patients with a relatively rare diagnosis.

## Conclusions

This study compares ambulation, urinary, and surgical outcomes in closed SB patients with a sac (> 3 mL) compared to no sac (≤ 3 mL) in a single high-volume fetal and pediatric neurosurgical center. We observed that patients with a sac had a significantly increased risk of prenatal talipes. At 5 years of age, patients with a sac also had a decreased ability to ambulate independently, increased use of CIC, and increased need for repeat tethered cord release compared to patients without a sac. Given the array of associated morbidities reported in this study, longitudinal multidisciplinary follow-up is critical in the care of these patients, especially those with a sac. As prenatal imaging and fetal diagnostic tools continue to improve, more research is needed to determine additional prenatal characteristics that can aid in the prediction of long-term outcomes to help advise care and to identify additional interventions to continue to improve outcomes for patients with closed SB.

## Data Availability

No datasets were generated or analysed during the current study.
